# Action observation training for rehabilitation in brain injuries: a systematic review and meta-analysis

**DOI:** 10.1186/s12883-019-1533-x

**Published:** 2019-12-27

**Authors:** Bianca Buchignani, Elena Beani, Valerie Pomeroy, Oriana Iacono, Elisa Sicola, Silvia Perazza, Eleonora Bieber, Hilde Feys, Katrijn Klingels, Giovanni Cioni, Giuseppina Sgandurra

**Affiliations:** 1 Department of Developmental Neuroscience, IRCCS Fondazione Stella Maris, Viale del Tirreno 331, 56128 Calambrone, Pisa, Italy; 2 Acquired Brain Injury Rehabilitation Alliance, School of health Sciences, University of East Anglia, Research Park, Norwich, NR31 9HL UK; 3 Department of Rehabilitation Sciences, KU Leuven - University of Leuven, Leuven, Belgium; 4 Rehabilitation Research Center, Faculty of Rehabilitation Sciences, Hasselt University, Diepenbeek, Belgium; 5 Department of Clinical and Experimental Medicine, University of Pisa, Pisa, Italy

**Keywords:** Brain damage, Mirror neurons, Neurological rehabilitation, Upper limb, Lower limb

## Abstract

**Background:**

To systematically review and analyse the effects of Action Observation Training on adults and children with brain damage.

**Methods:**

Seven electronic databases (Cochrane, EBSCO, Embase, Eric, PubMed, Scopus and Web of Science) were searched up to 16 September 2018 to select Randomized Controlled Trials focused on adults and children with brain damage that included AOT training on upper and/or lower limb carried out for at least 1 week. Identification of studies and data extraction was conducted with two reviewers working independently. Oxford Centre for Evidence-based Medicine (March2009) – Levels of Evidence and Physiotherapy Evidence Database scale were used to grade studies. The data collected from the articles were analysed using software R, version 3.4.3. Hedge’s g values were calculated and effect size estimates were pooled across studies. Separate meta-analyses were carried out for each ICF domain (i.e. body function and activity) for upper and lower limb.

**Results:**

Out of the 210 records identified after removing duplicates, 22 were selected for systematic review and 19 were included in the meta-analysis. Thirteen studies included in the meta-analysis focused on upper limb rehabilitation (4 in children and 9 in adults) and 6 on lower limb rehabilitation (only studies in adults). A total of 626 patients were included in the meta-analysis. An overall statistically significant effect size was found for upper limb body function (0.44, 95% CI: [0.24, 0.64], *p* < 0.001) and upper limb activity domain (0.47, 95% CI: [0.30, 0.64], *p* < 0.001). For lower limb, only the activity domain was analysed, revealing a statistically significant overall effect size (0.56, 95% CI: [0.28, 0.84], *p* < 0.001).

**Conclusions:**

Action Observation Training (AOT) is an innovative rehabilitation tool for individuals with brain damage, which shows promising results in improving the activity domain for upper and lower limbs, and also the body function domain for the upper limb. However, the examined studies lack uniformity and further well-designed, larger controlled trials are necessary to determine the most suitable type of AOT particularly in children.

**Systematic review registration:**

CRD42019119600.

## Introduction

Action observation therapy (AOT) is a novel rehabilitation strategy for both adults and children. It involves observation of meaningful actions with the intention to imitate and then performing those actions. AOT is based on neurophysiological knowledge that observation of a goal-directed action [1, 2] activates the same neural substrate, called the Mirror Neuron System, as does the physical execution of the observed action.

AOT has been investigated for its potential benefits for children with cerebral palsy (CP) [3–5], adult stroke patients [6, 7], individuals suffering from Parkinson’s [8] and Alzheimer’s disease [9]. The use of AOT in rehabilitation programs may have top-down effects involving higher-level networks that impact peripheral circuits, e.g. central movement planning areas, motor areas and peripheral structures [10]. To our knowledge, only few systematic reviews have explored AOT effectiveness on upper and lower limb rehabilitation. One was carried out in both neurological and orthopedic diseases [11]. Another was carried out on patients with stroke and explored AOT enhancement in motor function and upper limb motor performance [12]. Others have only explored the effectiveness of AOT on limb pain [13] and in Parkinson disease [8].

However, in previous reviews, no meta-analysis including studies on children and on lower limb was carried out. Moreover, the data were not analyzed taking account of the International Classification of Functional Disability and Health (ICF) framework. The ICF, with its multidimensional nature, provides an international framework for measuring and documenting health outcomes at the body function and structure level as well as for activities and participation.

This review addresses clinical research questions related to: i) how many studies focused on the rehabilitation of the upper or lower limb, ii) how many studies were conducted on adults and on children, iii) what type of training was conducted, where did it take place, how long did it last and did it influence the outcome, iv) what are the effects of AOT on upper and lower limb measures according to ICF domains, in adults and children with brain damage.

## Methods

### Design

A systematic review and meta-analysis following the guidelines of Preferred Reporting Items for Systematic Reviews and Meta-Analyses (PRISMA) [14] were conducted. Identification of studies that met the review criteria, assessment of methodological quality and data extraction was undertaken by two reviewers (BB, EB), working independently. Any disagreements were resolved through consensus or, when necessary, by a third reviewer (GS).

This systematic review was registered on PROSPERO (CRD42019119600).

### Inclusion and exclusion criteria (review criteria)

The criteria used to select articles were: i) participants were children or adults with brain damage; ii) investigated AOT training on upper and/or lower limb that was carried out for at least for 1 week; iii) randomized controlled trials.

The exclusion criteria were: i) articles written in languages unknown to the authors (i.e. Chinese, Persian); ii) participants with Parkinson’s disease; iii) reports in the form of abstracts, reviews, theses or conference papers; iv) AOT carried out with only the observational element and not followed by action and v) grey literature.

The literature search was conducted using seven electronic databases: PubMed, EBSCO, Cochrane, Scopus, Web of Science, Embase and Eric. The search dates were from database inception to 16th September 2018. The search used the following terms: (“Brain injury” OR “cerebral injury” OR “cerebrum lesion” OR “left hemisphere injury” OR “right hemisphere” OR “brain damage” OR “brain lesion” OR “stroke” OR “cerebral palsy” OR hemipleg*) AND “action observation” AND (“training” OR “treatment” OR “trial”).

### Identification of relevant articles

Two reviewers (BB, EB), independently, screened the titles and abstracts of identified articles. Duplicates were removed. All articles that probably or possibly fulfilled the study criteria were taken forward for full text screening. Each reviewer, again working independently, then examined the full text of articles to assess whether they met the study criteria. All articles that met the study criteria were included in this systematic review.

### Assessment of methodological quality

The methodological quality of the included studies was assessed according to the latest versions of: the Oxford Centre for Evidence-based Medicine (CEBM, March 2009) – Levels of Evidence [15] and the Physiotherapy Evidence Database scale [16]. Reviewers worked independently as described above.

### Data extraction

The two reviewers independently recorded for each included study: CEBM level; PEDro score; study aim/s; diagnosis; sample size; mean age of participants; setting; duration and intensity of training; type of AOT; video perspectives; other treatments provided to the experimental group; and the type of intervention and other concurrent treatments provided for the control group. Data was dichotomized by section (upper or lower limb) because different aims and outcome measures were used. Outcome measures regarding upper and lower limb were divided into ICF domains. If an outcome measure involved more than one domain, the outcome measure was classified within the most representative domain [17].

### Meta-analyses

Study outcome measures, results and findings of examined studies were analysed. The data collected from the articles were analysed using software R, version 3.4.3. Hedge’s g values were calculated and, according to Cohen [18], values of effect sizes between 0.2 and 0.5 were considered “small”, between 0.5 and 0.8 “medium”, and > 0.8 “large”.

Effect size estimates were pooled across studies to obtain an overall effect size. Some of the studies included different outcomes, that could be correlated [19]. A multivariate random-effect linear model was used to conduct a meta-analysis, where covariance matrix was explicitly provided to the model. Separate meta-analyses were carried out for each ICF domain (i.e. body function and activity) for upper and lower limb.

## Results

### Identification of included studies

**Fig. 1:**
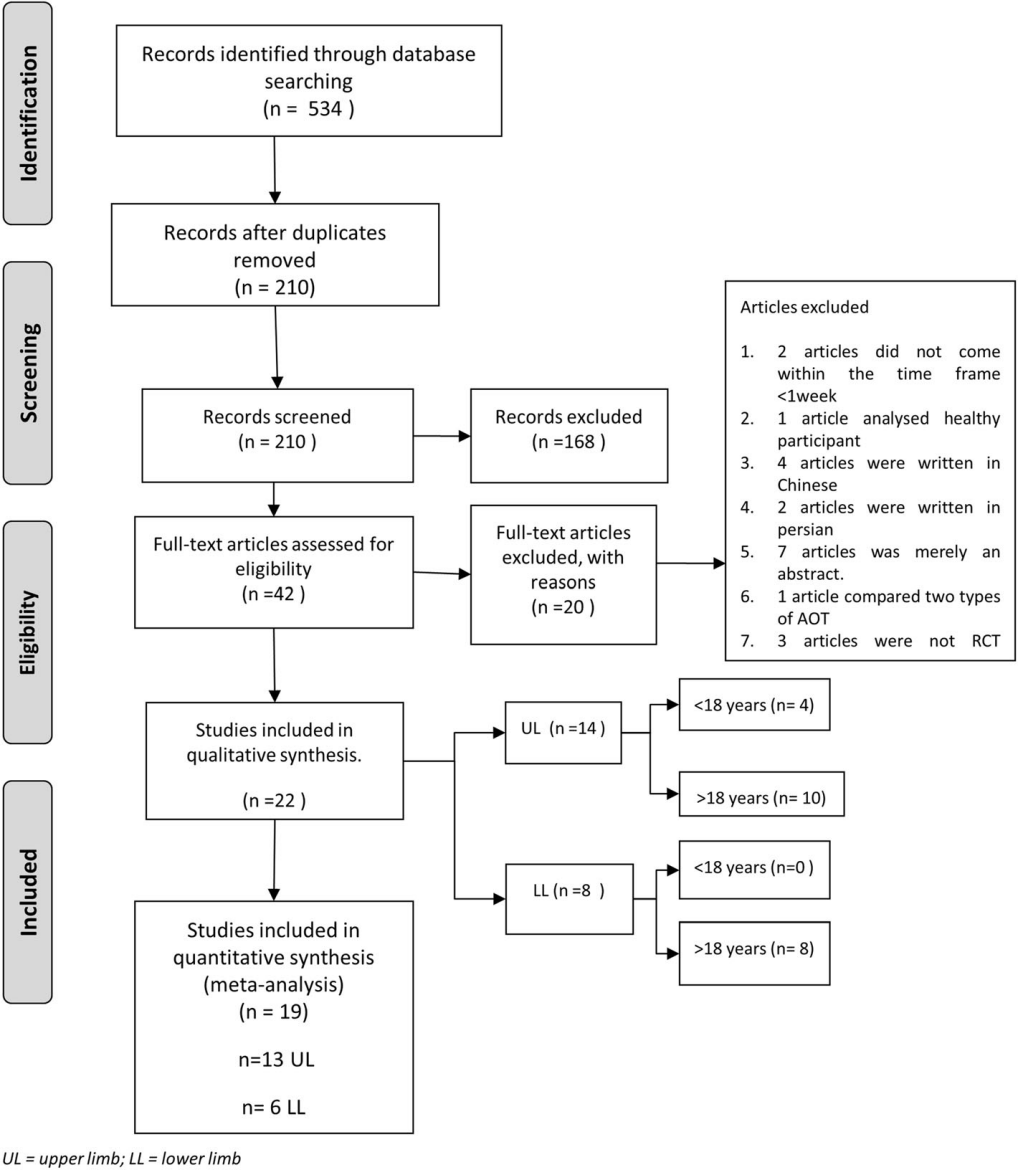
PRISMA Flow Diagram

The database search identified 534 articles, of which 210 remained after duplicates were removed. Of these, 168 records were excluded after the titles and abstracts were screened. Of the 42 full papers that were read 20 did not meet the inclusion criteria. The following studies were excluded: i) two studies had AOT training of less than 1 week; ii) one paper included healthy participants; iii) four articles were written in Chinese and two in Persian; iv) seven articles were merely abstracts; v) one article compared two types of AOT; vi) three articles were not RCTs. Consequently, 22 studies were included in this review (Fig. 1).

### Characteristics of included studies

The characteristics of the included studies are summarized in Tables 1, 2, 3 and 4. Of the 22 selected studies 14 focused on upper limb [1, 3, 5, 6, 20–29] and eight on lower limb rehabilitation [30–37]. In four studies, the participants were children [3, 5, 20, 22].

**Table 1:** Description of studies on upper limb rehabilitation (First part)

Author	CEBM level	PEDro Score	Diagnosis	Sample size		Duration (wks)	Intensity (Frequency per week, Minutes/day)
				Enrolled: tot;	Age (Mean +/− standard deviation) years		
Buccino G. et al.; 2018 [20]	1b	9/10	CP	18	5 to 11	3	5 session per week, 30 min each session
Fu J et al.; 2017 [21]	1b	7/10	Sub-acute stroke	53	62.04 +/− 9.93 (AOT group); 59.76 +/− 10.57 (control group)	8	6 times/week, 20 min/day
Kirkpatrick E et al.; 2016 [22]	2b	6/10	UCP	70	3 to 10	12	5 sessions per week, 15 min each session
Kim CH et al.; 2016 [23]	1b	9/10	Sub-acute stroke	22	62.78 +/− 9.85 (AOT group); 61.49 +/− 8.64 (control group)	4	5 times/week, 40 min/day
Zhu M-H et al.; 2015 [1]	1b	7/10	Stroke	61	42–75	8	6 times/week, 30 min/day
Kim E et al.; 2015 [24]	2b	3/10	Stroke	12	n.s.	6	5 sessions per week, 30 min per day
Kim E et al.; 2015 [25]	2b	3/10	Stroke	12	n.s.	6	5 sessions per week, 30 min per session
Sale P et al.;2014 [26]	1b	8/10	Subacute ischaemic stroke	67	66.50 ± 12.70	4	5 days/week, two 15-min daily session at least 60-min interval apart
Sgandurra G et al.; 2013 [5]	1b	8/10	UCP	24	5–15	3	15 consecutive working days, 60-min (including the rest periods) rehabilitation sessions
Lee D et al.; 2013 [27]	2b	5/10	Chronic stroke	33	63 ± 3.70 (Observation) 62 ± 1.50 (Action practice) 61 ± 2.30 (Combined) 60 ± 5.90 (control)	3	5 sessions per week, 10-min / day
Cowles T et al.; 2013 [28]	1b	7/10	Early after stroke	29	60–89	3	Each day for 15 working days, two 30 min sessions (approximately 6- to 8-min periods divided by 2 to 4 min of resting), separated by a 10 min rest
Buccino G et al.;2012 [3]	1b	7/10	CP (12 UCP 3 bilateral CP)	15	mean age = 6.80	3	5 times per week, 15 min/day
Franceschini M. et al.;2012 [29]	1b	7/10	Stroke	102	n.s.	4	5 sessions per week; 20 sessions (15 min =3 min sequence observations and 2 min action performances for 3 sequences); each session repeated twice a day, at least 60 min
Ertelt D. et al.; 2007 [6]	2b	6/10	Stroke	16	38–69	4	18 consecutive working days, 18 sessions of 90 min each

*n.s*. Not specified, *CP* Cerebral palsy, *UCP* Unilateral cerebral palsy

**Table 2:** Description of studies on upper limb rehabilitation (Second part)

Author	Experimental group		Control group	Study Outcome	Results: differences between experimental and control group
	Type of AOT	Videos (perspectives)	Type of intervention		
Buccino G. et al.; 2018 [20]	15 videos of task of daily action subdivided in ¾ motor segments	Different perspectives	Videos with no specific motor content + execution of same actions at the EG	MUUL, AHA	After treatment, the functional score gain was significantly different in the EG and CG
Fu J et al.; 2017 [21]	Observation (10 min) + imitating (10 min) Actions: Many different movements in different directions with different complexity	Each action was filmed from 2 different angles	Observe videos of different geometric patterns and symbols + performed action as the EG	FMA, WMFT, MBI, MEP	FMA, WMFT, MBI increased significantly compared with that before therapy in both groups. The indexes were significantly higher in the EG group compare to the CG
Kirkpatrick E et al.; 2016 [22]	watch a parent perform a movement + execution	(no video) egocentric viewpoint	Purposeful action observation program (without the observation)	AHA, MA2, ABILHAND-Kids	no between-group differences in AHA, MA2, or ABILHAND-Kids at 3 and 6 months vs baseline
Kim CH et al.; 2016 [23]	Observation (9 min), followed by a break (1 min to organize + practicing (30 min) Actions: Task-oriented training consisted of performing task based on ADLs	Each video provided 3 views simultaneously: front, side and top	The same tasks during a 30 min period, without watching the video	FMA, BBT, MBI, MAS	The mean change of FMA, BBT, and MBI in the AOT was significantly different between groups. No differences at MAS
Zhu M-H et al.; 2015 [1]	Observation followed by the action Actions: total of 30 actions showing many different movements or more complex actions	Straight on (20s), right above (15 s) and right inside (15 s)	Routine rehabilitation treatment and nursing	FMA, MBI, MAS	FMA, BI and MAS scores were significantly better after treatment in the EG compared to the CG
Kim E et al.; 2015 [24]	Observation of 2 from a variety of activities per session selected by patients, repeated over 1 week	n.s.	Perform the purposeful AO program without observing actions	WMFT	The EG showed significantly greater improvement compared with the CG
Kim E et al.; 2015 [25]	Observation of 2 daily life activities per session selected by patients repeated over 1 week	n.s.	Perform the AOT assignments, without the observational part	3D motion analysis system	EG showed improvement than the CG (no significant). Both groups showed improvements in average velocity, trajectory ratio, and movement degree, but not statistically significant
Sale P et al.;2014 [26]	Observation followed by performing the same tasks (2 min) from the easiest to the most complex action Actions: 20 daily activities composed by 3 different meaningful motor sequences displayed in order of ascending difficulty	first-person	Control Treatment: 5 static images displaying objects, without any animal or human being, for 3 min + to perform the same tasks of the EG	BBT, FMA	Significant higher gain for EG than CG, with respect to functional measures taken at T1 and T2. Left hemiparetic subjects achieved significantly greater benefits compared to the right ones. FMA and BBT between groups, statistically significant differences only for left hemiparetic.
Sgandurra G et al.; 2013 [5]	15 sets of daily life, un- or bi-manual goal-related actions of increasing complexity	First-person perspective	To watch computer games + verbally instructions to perform same actions as AOT group	AHA, MUUL, ABILHAND-Kids	Significant AHA within-group differences at all follow-up assessments. At T1 significant between-group difference and at T2 and T3 at the limits of significance. No differences at MUUL and ABILHAND-Kids
Lee D et al.; 2013 [27]	AOT group: observation of task video of drinking behaviour (5 min) followed by the actions (5 min)	From the front of the model	Observation group: observation of a task video (20 times); APG: repeatedly practiced actions performed during the preliminary test for 10 min; CG: neither watched the video nor practiced the actions	Number of times the full drinking action was performed in 1 min	All groups showed statistically significant improvements compared to CG. Combined group had a significant higher number of drinking behaviors than Observation group, immediately after and 1 week after the experiment. No statistical differences between the Combined and the AOT group
Cowles T et al.; 2013 [28]	Observation (1–2 min) followed by action (4–6 min) performed simultaneously with the therapist	No video	CPT as deemed appropriate	MI, ARAT	The median (95% CI) between-group difference was not statistically significant
Buccino G et al.;2012 [3]	motor tasks of actions related to the children’s daily lives	Different perspectives	Video (no specific motor content) + execution of same actions as the EG	MUUL	After treatment, the functional score gain was significantly different in the EG and CG
Franceschini M. et al.;2012 [29]	Observation of 1 task per day consisting in three different 3-min meaningful motor sequences, from the easiest to the most complex action + to imitate the observed motor sequence. The actions were 20 daily activities	first-person	Control treatment or “sham” AO = to observe for 5 min 5 static images (no motor content) + to perform UL movements as well as feasible for 2 min according to a standard sequence, simulating those performed by the EG	BBT, FAT, FMA, MAS, FIMM	Differences between the 2 groups were found from T0 to T1 and from T1 to T2. However, no difference was found on either change in BBT performance from T1 to T2. No significant difference between the study groups was found in the FIMM and FMA performance
Ertelt D. et al.; 2007 [6]	6 min videos showing daily life hand/upper limb actions + 6 min of repetitive practice of the observed actions with their paretic UL. 3 hand/upper limb movements of increasing complexity each day	3 different perspectives	Same as the EG but sequences of geometric symbols and letters. The practiced hand and upper limb actions were performed by instruction of the therapist in the exact order as they were practiced in the experimental condition	FAT, WMFT, SIS	Significant improvement of motor functions as compared to T0, and compared with CG, maintained for at least 8 weeks after the end of the intervention. Neural activations between EG and CG after training shows significant rise in bilateral ventral premotor cortex, bilateral superior temporal gyrus and supplementary motor area

*AOT* Action Observation Therapy, *EG* Experimental Group, *CG* Control Group, *MUUL* Melbourne Assessment of Unilateral Upper Limb Function Scale, *AHA* Assisting Hand Assessment, *FMA* Fugl-Meyer Assessment, *WMFT* Wolf Motor Function Test, *MBI* Modified Barthel Index, *MEP* Motor Evoked Potential, *MA2* Melbourne Assessment 2, *ADLs* Activities of Daily Living, *BBT* Box and Block Test, *MAS* Modified Ashworth Scale, *BI* Barthel Index, *n.s* not specified, *APG* Action Practice Group, *CPT* Conventional Physical Therapy, *MI* Motricity Index, *ARAT* Action Research Arm Test, *AO* Action Observation, *FAT* Frenchay Arm Test, *FIMM* Functional Independence Measure Motor Item, *UL* Upper Limb, *SIS* Stroke Impact Scale

**Table 3:** Description of studies on lower limb rehabilitation (First part)

Author	CEBM level	PEDro Score	Diagnosis	Sample size		Duration (wks)	Intensity (frequency per week, minutes/day)
				Enrolled tot;	Age (Mean +/− standard deviation) years		
Kim JC et al.; 2017 [30]	1b	8/10	Chronic stroke	21	57.08 ± 7.29 (AOPT group); 52.92 ± 8.21 LIOPT (control group)	3	3 days/week, 15 min × 2 /day;
Bae S et al.; 2017 [31]	1b	7/10	Chronic stroke	18	49.50 ± 10.60 (DASI); 49.67 ± 8.78 (control group)	4	5 days /week, 20 min day
Park HJ et al.; 2017 [32]	1b	7/10	Chronic stroke	25	57.33 ± 6.89 AOT group; 55.08 ± 8.12 control group	4	3 sessions per week, 30 min for video
Lee et al.; 2017 [33]	2b	5/10	Chronic stroke	35	62.80 ± 7.40 (AOTA group); 57.27 ± 5.70 (MTA group) 59.80 ± 6.70 (AOT group)	6	3 times per week, 30 min/day
Park and Hwangbo; 2015 [34]	2b	4/10	Chronic stroke	40	51.15 ± 14.81 AOGT; 48.65 ± 12.81 GGT;	8	5 times per week, 30 min per session
Park HR et al.; 2014 [35]	1b	7/10	Chronic stroke	21	55.91 ± 9.10 (AOT group); 54.80 ± 12.22 (control group)	4	3 times per week, 30 min/day
Kim JH et al.; 2013 [36]	2b	6/10	Chronic stroke	27	55.30 ± 12.10 AOT group; 54.80 ± 8.80 MIT group; 59.80 ± 8.90 PT group	4	5 times / week, 30 min for session
Kim JH et Lee BH; 2013 [37]	2b	6/10	Chronic stroke	27	55.30 ± 12.10 AOT group; 54.80 ± 8.80 MIT group; 59.80 ± 8.90 PT group.	4	5 times / week, 30 min for session

*EG* Experimental Group, *CG* Control Group, *LIOPT* Landscape Imagery Observation Physical Training Group, *AOPT* Action Observation Physical Training Group, *DASI* Dual-Afferent Sensory Input, *FES* Functional Electric Stimulation, *AOTA* Action Observation Therapy with Activity, *MTA* Mirror Therapy with Activity, *AOGT* Action Observation Gait Training Group, *GGT* General Gait Training Group, *AOT* Action Observation Therapy, *MIT* Motor Imagery Group, *PT* Physical Training, *min* Minutes

**Table 4:** Description of studies on lower limb rehabilitation (Second part)

Author	Experimental group		Control group	Study Outcome	Results: differences between experimental and control group
	Type of AOT	Videos (perspective; speed)	Type of intervention		
Kim J-C et al.; 2017 [30]	Observation (2 min 30 s) + 12 min 30 s for physical training × 2/day Actions: tasks related to STW and imitated actions. 16 tasks with adjusted difficulty and condition based on patient’s functional status and level	n.s.	Observe static landscape photos + physical training as the EG	WDI, LOS, TUG, DGI	No significant difference in the TUG, DGI, and WDI between the AOPT and LIOPT groups. Significant difference in LOS between the AOPT and LIOPT groups
Bae S et al.; 2017 [31]	20 min. Video of dorsiflexion of the contralateral ankle recorded in advance whit simultaneously application of ETFES, movement of the contralateral ankle, induced by ETFES shown live on a monitor during subjects’ performance	n.s.	Patients were instructed to dorsiflex upon FES application. A Microstim device was used to apply FES by bipolar placement of the electrodes. Asymmetrical biphasic waves were applied for 20 min with valgus position	MRCP was measured by the QEEG-8; the H reflexes with Neuro-EMG-Micro, EMG, and Biorescue system for assessment of the effects of ETFES with AOT	MRCP in MP at C4 and dynamic balance (LOS) showed significant differences between DASI and control group
Park HJ et al.; 2017 [32]	video clips of walking on even and uneven ground, in a complex and unpredictable community environment, in a parking lot, shopping center	3 different directions (front back, side), 2 different filming speeds: normal and half times normal speed.	30 min video clips of static landscape pictures; any human or animal representation were excluded	10MWT	In EG walking function and ambulation confidence was significantly different between the pre- and post-intervention, whereas the CG showed a significant difference only in the 10MWT
Lee et al.; 2017 [33]	Observation (15 min) + execution (15 min) Actions: dorsiflexor training composed of 3 stages of active assistive exercise. 1 stage: knee joint extensor and dorsiflexor training. 2 stage: knee joint flexor and dorsiflexor training. 3 stage: hip and knee joint flexor and dorsiflexor training	Front and lateral side videos were produced separately for the left and right hemiplegic subjects	The MTA group received mirror therapy for 15 min/day and physical training of the same motions without a mirror for 15 min/day. The AOT group conducted action observation only for 30 min/day	OBI, ABI, MBI, Postural stability and fall risk, mEFAP	No significant difference was found between the groups on all outcome measures
Park and Hwangbo; 2015 [34]	AOGT: 3 min video+ 1 min break + 5 min walking training + 1–2 min break. (x3)	n.s.	GGT: 12 min video with break (3 min) showing images of nature unrelated with walking + 20 min walking training	Balance ability: sway area, sway speed, limit of stability by analysis system using biofeedback, AP1153BioRescue. Gait ability: TUG, 10MWT	There were significant differences in the sway speed, in the limit of stability, in TUG and 10 MWT between the two groups after the experiment but not in the sway area
Park HR et al.; 2014 [35]	Observation (10 min) of video clips + sessions of walking training (20 min). 4 Tasks for functional training frequently experienced in premorbid life including weight shifting to the affected side, walking on straight and curved paths, walking on even and uneven surfaces, crossing obstacle.	2 filming speed options (normal and half- speeds) in the front, back and side views in twice sequence	Observation of video clips showing different landscape images (10 min) + perform the same walking tasks as the EG	10MWT, DGI, Gait Symmetry Score	The difference between the pre- and post-test values of the 10MWT, figure-of-8 walk test, and DGI showed statistically significant differences between the EG and CG
Kim JH et al.; 2013 [36]	Observation (20 min) + Physical training with a therapist (10 min). Actions: 4 stages including trunk flexion, trunk rotation, sit to stand, and crossing obstacles.	n.s.	MIG: 20 min of motor imagery program played through a computer speaker + physical training for 10 min based on the training contents. PTG: training of the trunk for learning supine to rolling movements, sit to stand, and normal gait pattern	EEG data quantitative analysis using Telescan 2.9. Raw EEG data were converted into frequencies, then relative alpha power (8–13/4–50 Hz) and relative beta power (13–20/4–50 Hz) were analyzed	There were no significantly differences between the 3 groups
Kim JH et Lee BH; 2013 [37]	Observation of task video (20 min) + physical training with a therapist (10 min) Actions divided in 4 stages: Stage 1) pelvic tilting, trunk flexion and extension, and trunk rotation in the sitting position; Stage 2) sit to stand and stand to sit; Stage 3) weight shift to the front and back, left and right; Stage 4) gait level surface and step over obstacle	The video was produced separately for patients with left hemiplegia and right hemiplegia	MIG: 20 min of motor imagery program + physical training for 10 min as in the EG program. PTG: training of the trunk for learning supine to rolling movements, sit-to-stand, normal gait pattern, as well as training of the lower extremity, weight shifting, and gait level surface and gait stairs	TUG, the functional reaching test, the walking ability questionnaire, the functional ambulation category. Spatiotemporal gait parameters were collected using a GAITRite system	No significant differences in any outcome measure were observed between the AOT group and the MIG, except for Stride length. Significant difference was observed between the AOT group and the PTG in the TUG, gait speed, cadence, and single limb support of the affected side

*EG* Experimental Group, *CG* Control Group, *LIOPT* Landscape Imagery Observation Physical Training Group, *AOPT* Action Observation Physical Training Group, *STW* Sit To Walk, *PT* Physical Therapy, *WDI* Weight Distribution Index, *LOS* Limit of Stability, *TUG* Time Up and Go Test, *DGI* Dynamic Gait Index, *DASI* Dual-Afferent Sensory Input, *FES* Functional Electric Stimulation, *EMG* Electromyography, *ETFES* Electromyography triggered-functional electric stimulation, *TA* Tibialis Anterior, *MRCP* Movement-related cortical potential, *MP* Motor Potential, *10MWT* 10 Meter Walk Test, *MTA* Mirror Therapy with Activity, *OBI* Overall Balance Index, *ABI* Anteroposterior Balance Index, *MBI* Mediolateral Balance Index, *mEFAP* Modified Functional Ambulation Profile, *AOGT* Action Observation Gait Training, *GGT* General Gait Training, *F8W* Figure of 8 walk test, *MIG* Motor Imagery Group, *PTG* Physical Training Group, *n.s*. not specified

Sample sizes ranged from 12 [24, 25] to 102 [29]. All participants had a clinical diagnosis of stroke or cerebral palsy (CP).

AOT was undertaken in several ways. In most studies, videos with the performed actions were shown [1, 3, 5, 6, 20, 21, 23, 26, 27, 29–37]. In two studies, a therapist or the mother performed the action [22, 28]. Settings were a laboratory and in-patient hospital environment [1, 3, 5, 6, 20, 21, 23–30] except for one study [22], where the setting was the participants’ homes. The setting was not specified in two articles [21, 31].

In many studies, control groups watched videos in which no action was shown [3, 5, 6, 26, 29, 30, 32, 34, 35], while in other studies, an action was performed without an observation phase [22–25, 27, 28]. In one study, the control group was provided with routine rehabilitation [1].

The duration of AOT ranged from 3 weeks [3, 5, 20, 27, 28] to 12 weeks [22]. The amount of AOT ranged from 10 min a day [27] to 90-min a day [6]. The mean ± SD duration was 4.91 ± 2.31 weeks and the mean ± SD amount 32.05 ± 17.84 min.

Three ICF domains (body function, activity and participation) were assessed across the different studies, even if in each domain different outcome measures were often used. Only few studies used the same outcome measures in body function or activity domain (e.g. Fugl Meyer Assessment (FMA) [1, 21, 23, 26], Melbourne Unilateral Upper Limb Assessment (MUUL) [3, 5, 20], Box & Block Test (BBT) [23, 26, 29], Assisting Hand Assessment, (AHA) [5, 20, 22]).

### Quality indicators

CEBM level was applied in all studies and 12 studies were classified at level 1b [1, 3, 5, 20, 23, 26, 28–32, 35], 9 at level 2b [6, 21, 22, 24, 25, 27, 33, 34, 36, 37]. PEDro scale results are shown in Tables 1 and 3. PEDro scores ranged from 3 to 9; most studies obtained 7/10 [1, 3, 28, 29, 31, 32, 35], only two studies scored 9/10 [20, 23].

### Studies focused on upper limb

#### Sample participants

Studies on upper limb were carried out on very heterogeneous samples. Two studies included chronic stroke patients (> 6 months duration) [6, 27]; two enrolled only patients with first-ever stroke, 30 days (±7) after the onset of the event with ischemia or primary haemorrhage [26, 29]; one study included adults who had suffered a stroke 3 to 31 days prior to recruitment [28]; and the remaining three studies [1, 21, 23] enrolled subjects within 6 months of stroke. Two studies did not specify whether patients were in their subacute or chronic post-stroke phase [24, 25]. Moreover, regarding studies on children, two focused on children with Unilateral Cerebral Palsy (UCP) [5] while children with unilateral and bilateral CP were included in two other studies [3, 20]. It is important to highlight that all children with CP had a cognitive level within normal limits for verbal functions and did not present any sensory impairments [3, 5, 20, 22].

#### AOT training and control conditions

Videos showing various actions [1, 23, 27] or videos of daily routines [6, 26, 29] were used in the experimental group. Actions demonstrated in two studies [26, 29] were both unimanual and bimanual. In four studies [1, 6, 21, 26], the difficulty of the proposed actions increased incrementally during treatment. Participants performing the action on the video were healthy men or women in four studies [20, 23, 26, 29].The type of model performing the action was not explicitly mentioned in the others. In one study [5]), separate videos were produced for patients with left or right hemiplegia.

Some of the studies specify the perspective from which actions were performed. In one study [23] three perspectives were provided simultaneously: front, side and top. In another study [1], actions were seen from “straight on, right above and right inside”, whereas in yet another [27] the video was shot from the front. In three studies [5, 26, 29] actions were observed from a first-person perspective. Three [6] reported that actions had been recorded from different perspectives (one [6] specified that 3 perspectives had been used) but failed to mention which ones, and in another two, no mention was made at all of the type or number of perspectives [24, 25]. Two studies did not use videos but life demonstration to show actions [22].

In the home-based study [22], a parent performed the action while sitting next to the child on the less-affected side facing in the same direction, so that the child observed the hand movements from an egocentric viewpoint, whereas in the in-patient study [28] the therapist sat next to the participant on his/her affected side, demonstrating the action to be performed.

Control groups performed actions without observation [23–25], or they observed videos, images, or sequences of geometric symbols [6] which showed a neutral environment [26, 29] and performed the same actions as the experimental group. In one study, children were asked to play computer games [5].

In all the studies where AOT observation phase was conducted using videos a significant change in at least one outcome measure was found. On the contrary, the only two studies [22, 28] where, instead of the videos, patients observed a person performing an action, reported no significant functional improvements, neither in adults with stroke in an early phase (mean 18.70 days) [28] nor in children with UCP [22].

#### Duration of experimental and control intervention

Duration of studies carried out on adults varied. Four studies lasted 4 weeks [6, 23, 26, 29], while most of the others were carried out over a 3-week period [3, 20, 22, 27, 28]. Two were 8 weeks [1, 21] long and the longest one lasted 12 weeks [22]. In all the studies, except two [1, 21], training took place 5 days a week.

Length of training sessions also varied, from a minimum of 10 min [27] to a maximum of 90 min [6]. Of the 10 studies examined, four were 30 min long [1, 20, 24, 28], four lasted 15 min [3, 22, 26, 29], one 40 min [23] and another lasted 60 min [5]. In two studies [26, 29] the session was repeated twice a day. The total intensity varied from a minimum of 150 min [27] to a maximum of 1440 min [1], however, in the majority of studies the total intensity was 900 min [5, 22, 24, 25, 28]; the overall mean ± sd of total intensity was 853.214 ± 410.78 min.

#### Outcome measures

In Table 5, various outcome measures are shown according to ICF domains. To investigate body function domain, all the four studies on children used the Melbourne Assessment [3, 5, 20, 22]. In adults five studies used the Fugl Meyer Assessment [1, 21, 23, 26, 29] or the modified Ashworth Scale (MAS) [1, 23]. A further study used the Motricity Index (MI) [28], while a kinematic analysis was carried out in one study [25].

**Table 5:** Upper limb outcome measures for each ICF domain in children (< 18 years) and adults (> 18 years)

	Upper limb outcome measures		
	Body function	Activity	Participation
Children	MUUL [3, 5, 20], MA2 [22]	AHA [5, 20, 22] ABILHAND-Kids [5, 22]	
Adults	FM [1, 21, 23, 26, 29] MAS [1, 23, 29] MI [28] Kinematic Analysis [25],	WMFT [6, 21, 24] BBT [23, 26, 29] FIMM [29], BI [1], MBI [21, 23] FAT [6, 29] Complete drinking actions [27], ARAT [28]	SIS [6]

*MUUL* Melbourne Assessment of Unilateral Upper Limb Function Scale, *MA2* Melbourne Assessment 2, *AHA* Assisting Hand Assessment, *BBT* Box and Block Test, *FM* Fugl-Meyer, *MAS* Modified Ashworth Scale, *MI* Motricity Index, *WMFT* Wolf Motor Function Test, *FIMM* Functional Independence Measure Motor Item, *BI* Barthel Index, *MBI* Modified Barthel Index, *FAT* Frenchay Arm Test, *ARAT* Action Research Arm Test, *SIS* Stroke Impact Scale

All the studies focused on ICF activity domain used various outcome measures. The same outcome measure was applied in a maximum of three studies. Three of the four studies on children had at least one ICF activity domain outcome measure i.e. Assisting Hand Assessment [5, 20, 22], ABILHAND-kids [5, 22]. Three studies in adults used the Box and Block Test [23, 26, 29], two used the Frenchay Arm Test [6, 29, 38] and three used the Wolf Motor Function Test (WMFT) [6, 21, 24]. Three studies also used the Barthel index (BI) [1, 21, 23] (in two articles [23] a modified version was used). Other outcome measures can be seen in Tables 3 and 4.

Only one study [6] analyzed participation domain using the Stroke Impact Scale (SIS) (see Table 5).

#### Meta-analysis of studies investigating AOT for upper limb rehabilitation

Of the 14 AOT studies on upper limb, only those with clinical standardized measures were included. For this reason one article [25] was excluded.

**Fig. 2:**
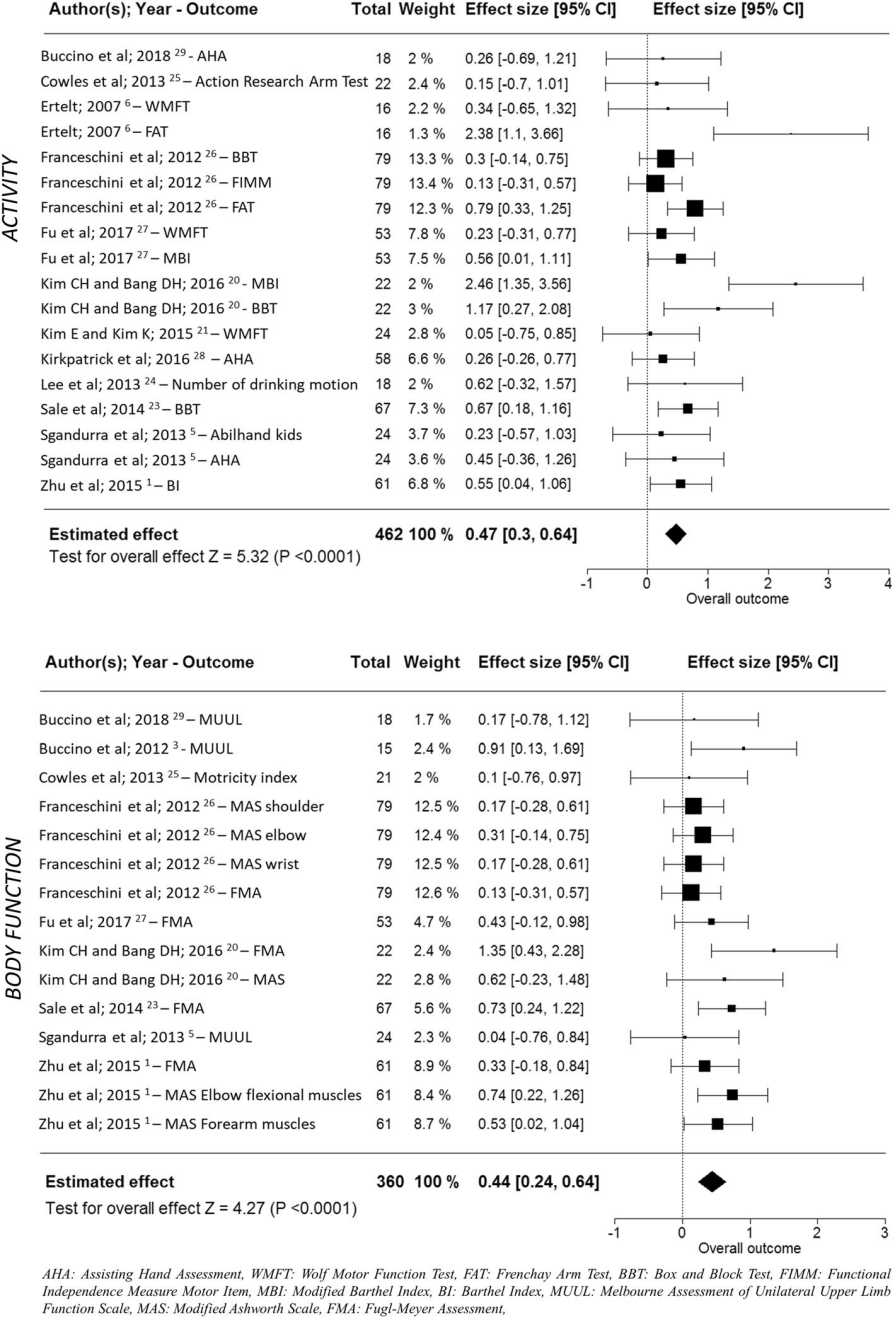
Meta-analysis of studies investigating AOT for upper limb rehabilitation

For body function domain, we analysed nine studies, including nine outcome measures on a total sample of 360 patients (169 allocated in AOT group). According to the multivariate random-effect model, overall effect size was statistically significant (*p* < 0.001), estimated as 0.44 (95% CI: [0.24, 0.64]) (Fig. 2).

Twelve studies were analysed in the activity domain, with 11 outcome measures and a total of 462 patients (219 allocated in AOT group). The multivariate random-effect model returned an overall statistically significant effect size (*p* < 0.001) of 0.47 (95% CI: [0.30, 0.64]).

### Studies focused on lower limb

#### Sample participants

All eight studies were carried out on adult patients diagnosed with chronic stroke.

#### AOT training and control conditions

All the studies on lower limb showed videos to the participants. Videoclips entailed walking in different locations and on different surfaces in two studies [32, 34], while an exercise of weight shift to the affected side was included in another [35]. In one study [30], participants observed Sit To Walk (STW) video tasks and imitated the actions. Action observation tasks consisted of 16 STW tasks in which difficulty and conditions were adjusted to patient functional status and level. In two studies [36, 37], several stages in the video included trunk flexion, trunk rotation, sit to stand, and stepping over obstacles to enhance balance and gait ability. In another one [33], there were three stages of an active assistive exercise: the first showed knee joint extensor and dorsiflexor training, the second knee joint flexor and dorsiflexor training and the third hip joint flexor training.

Generally, the models were healthy male/female adults. Separate videos were produced for patients with left or right hemiplegia in two studies [33, 37] . The perspective was specified only in two studies [33, 35] and speed of sequence was reproduced in fast and slow motion in the front, back and side views in one study [35]. The action was presented at normal speed and half the normal speed in another study [32].

The type of treatment offered to control groups, when present, varied: four groups watched videos showing static landscapes [30, 32, 35] or nature pictures not related to walking [34]. In one study, the control group [33] performed mirror therapy and physical training of the same movements of AOT, while in another, only action observation was conducted without any physical training. In two other studies [36, 37], where two control groups were present, one participated in a motor imagery program and did physical training similar to AOT group, while the other performed only physical training.

In one study [31], AOT was combined with electromyography-triggered functional electric stimulation (ETFES) in order to improve voluntary functional movement which was compared to training of subjects in a control group who underwent functional electric stimulation (FES).

#### Duration of experimental and control intervention

Most interventions lasted 4 weeks [31, 32, 35–37] while only one study lasted 6 weeks [33] and another lasted 8 weeks [34]. In four studies, sessions lasted 30 min and took place 3 times a week [30, 32, 33, 35]. In three studies, participants attended 30-min sessions, 5 times a week [34, 36, 37], whereas in one study patients attended a 20-min session 5 times a week [31]. The total intensity varied from a minimum of 270 min [30] to a maximum of 1200 min [34] the total intensity mean ± sd was 541.25 ± 292.79.

#### Outcome measures

When assessing lower limb rehabilitation, the main outcomes focused on body function and activity domains (Table 6).

**Table 6:** Lower limb outcome measures for each ICF domain in adults

	Lower limb outcome measures		
	Body function	Activity	Participation
Adults	Spatio-temporal gait parameters [32, 37] EMG [31] MRCP [31] H-reflex [31] balance parameters [33, 34] EEG [36] Weight Distribution Index [30] Limit Of Stability [30, 31]	ABC3 [2] FRT [37] 10MWT [32, 34, 35] TUG [30, 34, 37] figure of 8 walking test [35] dynamic gait index [30, 35] Community walking test [32] mE-FAP [33] WAQ [37] FAC [37]	

*EMG* Electromyography, *MRCP* Movement-related cortical potential, *10MWT* 10 Meter Walk Test, *TUG* Time Up and Go Test, *mEFAP* Modified Functional Ambulation Profile, *WAQ* Walking Ability Questionnaire, *FAC* Functional Ambulation Category, *ABC* Activities-specific Balance Confidence, *FRT* Functional Reaching Test

Balance was the most frequent outcome for the body function domain. However, this was assessed differently in four of the eight studies [30, 31, 33, 34] hampering comparisons of studies. In the ICF activity domain the most frequent measures referred to gait such as the TUG [34, 37] and 10MWT [32, 34, 35] (see Table 4).

#### Meta-analysis results on studies on lower limb rehabilitation

**Fig. 3:**
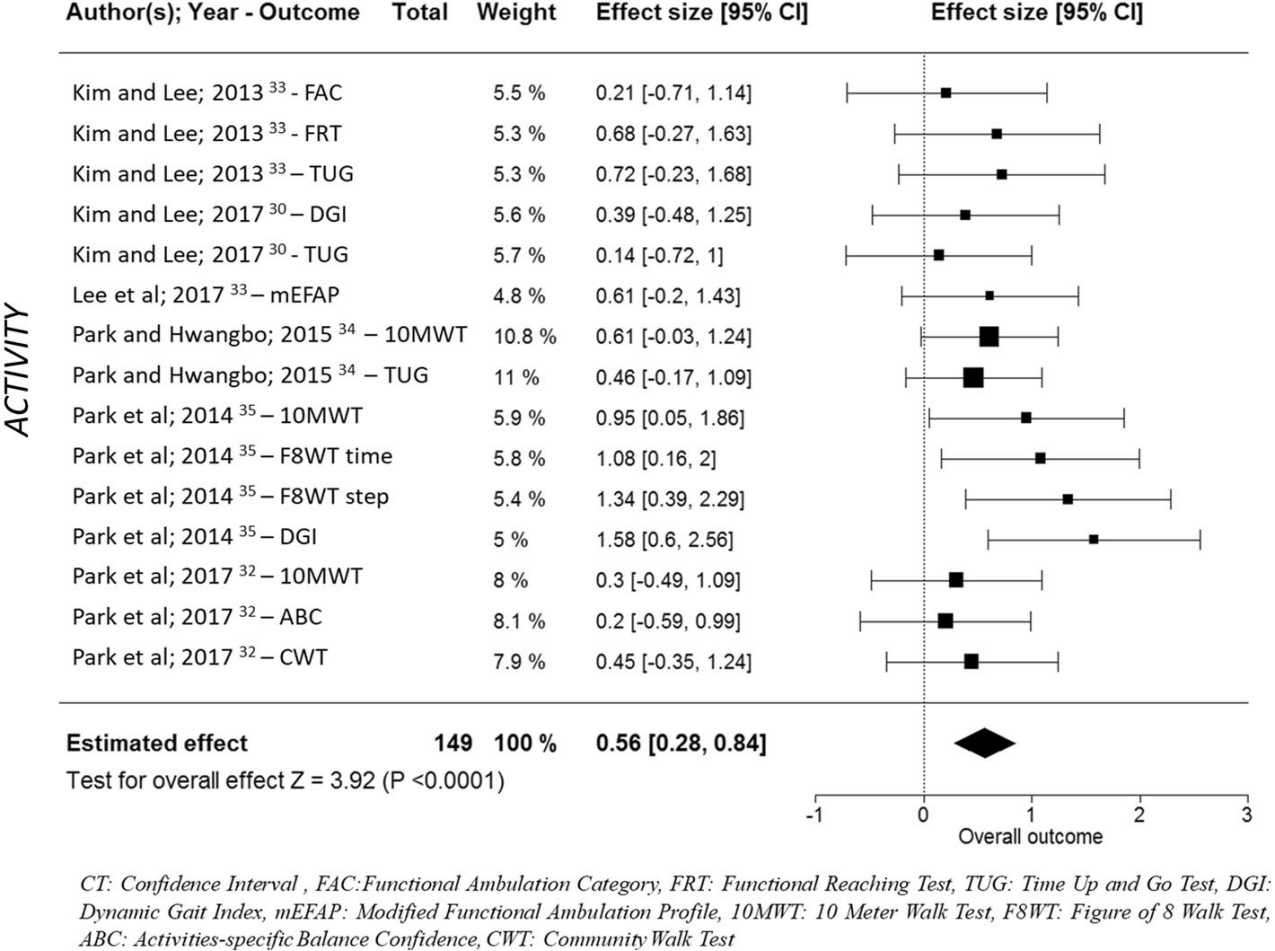
Meta-analysis of studies investigating AOT for lower limb rehabilitation

Of the eight included articles that focused on the lower limb, two articles [31, 36] were omitted from the meta-analysis because they did not use clinical standardized measures. All of the outcome measures for the lower limb across the six studies were in the ICF activity domain. The six studies used seven different outcome measures. In the multivariate random-effect meta-analyses, the overall effect size was statistically significant (*p* < 0.001), estimated as 0.56 (95% CI: [0.28, 0.84]) (Fig. 3).

## Discussion

### Main findings

Twenty-two RCTs with a total of 748 patients were included in this review. Of these 14 focused on upper limb and 8 on lower limb rehabilitation. Four studies were carried out in children, 18 in adults. The selected articles focused only on AOT as a rehabilitation tool in stroke and CP patients. There were no RCT studies using AOT training for other brain injuries such as multiple sclerosis or acquired or traumatic brain injury.

AOT observation phase was mainly conducted using videos and all these studies reported a significant change in at least one outcome measure. On the contrary, the only two studies [22, 28] where, instead of the videos, patients observed a person performing an action, reported no significant functional improvements, neither in adults with stroke in an early phase (mean 18.70 days) [28] nor in children with UCP [22]. Moreover, videos are both easier to standardize and allow a broader range of patients to benefit from therapy. However, a very recent pilot study on 12 children with CP [39] suggested that live AOT is more effective than video AOT. We did not include the study in the current review because there was no control group but two types of AOT were carried out and compared. Other studies with larger sample sizes and long-term follow up are necessary to identify which is the best approach.

From a qualitative analysis of data, the use of different perspectives in AOT videos did not allow us to understand whether some perspectives are better than other, nor to assess whether the type of perspective used is relevant. This could be related to the lack of standardization of the perspectives used or to other characteristics of the study. Given the variability of the perspectives used in videos, a standardization of these variables is needed to provide the most effective AOT.

One study [28] showed greater improvement in the control group than in the AOT for one outcome measure (Action Research Arm Test) which was used only in this study.

In this review, evidence of AOT effectiveness on motor functions, compared in the majority of the studies to physical therapy, was found on both children with CP and post-stroke adults. In the meta-analysis AOT significantly improves body function and activity domains with small and medium effect size for upper limb and lower limb, respectively.

Therefore, it could be hypothesized that the observation has a crucial add on effect to the motor activity, that is the main ingredient of conventional therapy.

### Comparison with early reviews

Comparing this review to the literature, there are two systematic reviews [11] [12] analysing AOT studies on patients with neurological diseases. The first review by Sarasso [11] also includes Parkinson’s and orthopedic diseases, while the review by Borges [12] is carried out on patients with stroke but focuses only on upper limb. This review adds seven articles [20, 22] on AOT in both children and adults to the previous ones. However, three articles included in the previous reviews were not acquired by our search strategy [40] and three studies did not match our inclusion criteria [41–43]: the training lasted less than a week in one articles [42], while the observation of the action was simultaneous to the practice in the other two [41, 43].

The most recent review [12] included studies up to September 2017 while our review involves studies up to September 2018.

The conclusion of the previous reviews [11, 12] suggested the efficacy of AOT in improving motor functions either in neurological and orthopedic diseases and of the upper limb in adults with stroke. Our findings corroborate and extend the previous ones. We were able to identify a larger number of studies in which AOT was used to rehabilitate not only the upper limb but also the lower limb of adults and children with brain injuries. The sample size of the present review, compared to the previous reviews, was the largest also including a meta-analysis for upper and lower limb. Moreover, we evaluated the effectiveness of AOT according to different ICF domains.

Comparing our results with the previous reviews, we also acknowledge the lack of dosage uniformity as highlighted in the previous reviews. Nonetheless, most studies lasted 3 to 4 weeks and sessions were about 30 min. However, even though a metanalysis comparing dose of treatment was carried out on upper limb by Borges [12],showing no significant difference, the attention span of children and adults should be considered when deciding duration and type of treatment.

It would also be useful to understand if there is a minimum threshold before an effect is produced on mirror neuron system and if a minimum duration is necessary to maintain the effect over time. Only some studies [5, 20, 22, 26, 27, 29, 31] have a follow-up assessment, so studies to understand the long-term effects after AOT are needed.

Regarding the differences between video and operator observation highlighted in the previous review, a further study [22] recorded no significant change in the outcome measures, in contrast with a recent article [39] which suggested that live AOT is better than video AOT. However, the articles enrolled small samples and further studies are needed. Finally, even though a recent review and multiple studies focused on Parkinson’s disease, the role of AOT in Parkinson’s rehabilitation is outside the aim of this review.

### Limitations of this review

The samples recruited in most RCTs were small (only five studies enrolled more than 50 patients [1, 21, 22, 26, 29] and studies adopted different inclusion and exclusion criteria, resulting in very heterogeneous populations. Moreover, in the studies on children, different types of CP were included. In addition to this, the sample selection was different, and this could affect the results of the papers and, thence, the finding of our work.

A potential limitation of this study is the risk of selection bias: the papers were identified through searches of selected databases, no reference lists of relevant papers were screened, no search for grey literature was conducted and papers published in Chinese and Persian were not included. These two issues are quite relevant because unpublished papers could have reported results in contrast with positive findings on the same topic, even if some studies included in the current review [22, 28] did not report significant results on the efficacy of AOT. In addition, we did not manage to translate papers not written in English language, which potentially can add information for the current review. In the future, if there will be a growing interest in conducting studies on AOT, an update of the present review could confirm or redefine the current findings.

### Strength of this review

We have analysed for the first time the effectiveness of AOT training in relation to ICF model, which is the most updated and international common framework for evaluating different disabilities, planning and measuring effects of different rehabilitation approaches. Moreover, the overall grade of recommendation based on CEBM model was A (i.e. consistent level-one study) since most studies reviewed were level one for both upper limb and lower limb.

### Recommendation for clinical practice

The studies on AOT are mainly carried out in the research field. However, they give insights for application in clinical practice. The AOT results suggest that the core of rehabilitation intervention should spent time in the observation of the activities before their execution. Moreover, the repetition of the motor activity should be followed by the observation of the proposed model in order that the patient can match the observation with his/her performance. The observation of a motor activity followed by practice can be easily applied by the therapists in the rehabilitation service as the he content of the exercises commonly provided in rehabilitation setting can be easily implemented in the framework of AOT focusing the rehabilitation in a more “top-down” perspective.

### Recommendation for future research

Future well-designed and sufficiently powered studies on AOT in brain injuries and multiple sclerosis have to be encouraged both in adults and even more in children. Larger scale studies should select homogeneous populations in children (e.g. AOT effect on a sample of patients with UCP, rather than a larger sample that included children with both unilateral and bilateral CP) and should investigate AOT effect on lower limb rehabilitation in children.

Moreover, thanks to the type of training which relies on the content of actions to be observed and on patient motivation to carefully observe to imitate and actively replicate the actions, AOT can be easily carried out at home. Well-standardized home-based studies need to be encouraged, as these would reduce not only hospital stays, travelling and waiting time for therapy, but would also allow for a much greater number of patients to benefit from this treatment. With this in mind, a recent trial [44] studies the effectiveness of home-based upper-limb AOT in children with UCP employing the latest technologies. However, further studies comparing different settings (e.g. hospital versus home) are needed. In addition, AOT video should be standardized as far as the perspective used (first-person or other) and length; moreover, mainly for children, the attention span should be considered when deciding duration and type of video and treatment.

Finally, larger controlled trials are necessary to determine the most suitable type of AOT regarding environment, treatment, control group and outcome measures in order to promote functional improvement of upper limb and lower limb, particularly in children.

## Conclusion

In conclusion, this is the first systematic review in which the effectiveness of AOT, separately both on the lower and upper limb function, is explored, also through a meta-analysis based on the ICF framework for the analysis of its efficacy. In particular, the findings are very promising, because data suggest the use of AOT for improving the activity domain for upper and lower limb, and also the body function domain for the upper limb. However, suitably powered RCTs on more homogeneous and larger samples, by means of valid and reliable paradigm and outcome measures, are required to confirm the real efficacy of AOT. A strong design comparing different lengths of AOT treatment and this novel approach with other types of rehabilitation is needed to demonstrate the specific role of AOT to replace or to be added to traditional rehabilitation.

## Data Availability

The datasets supporting the conclusions of this article are included within the article.

## Abbreviations

AHA: Assisting Hand Assessment
AOT: Action observation therapy
BBT: Box & Block Test
BI: Barthel index
CEBM: Centre for Evidence-based Medicine
CP: Cerebral Palsy
ETFES: Electromyography-Triggered Functional Electric Stimulation
FES: Functional Electric Stimulation
FMA: Fugl Meyer Assessment
ICF: International Classification of Functional Disability and Health framework
MAS: modified Ashworth Scale
MI: Motricity Index
MUUL: Melbourne Unilateral Upper Limb Assessment
PEDro: Physiotherapy Evidence Database scale
PRISMA: Preferred Reporting Items for Systematic Reviews and Meta-Analyses
RCT: Randomized Controlled Trial
SIS: Stroke Impact Scale
STW: Sit To Walk
UCP: Unilateral Cerebral Palsy
WMFT: Wolf Motor Function Test
